# Femur shaft fracture at a young age and the risk of subsequent severe injuries during childhood: a cohort study

**DOI:** 10.1186/1471-2431-14-62

**Published:** 2014-03-03

**Authors:** Johan von Heideken, Tobias Svensson, Maura Iversen, Anders Ekbom, Per-Mats Janarv

**Affiliations:** 1 Department of Women’s and Children’s Health, Karolinska Institutet, Karolinska University Hospital, Solna, SE 171 77, Stockholm, Sweden; 2 Department of Medicine, Solna, Clinical Epidemiology Unit, Karolinska Institutet, Stockholm, Sweden; 3 Department of Physical Therapy, Movement and Rehabilitation Sciences, Northeastern University, Boston, Massachusetts; 4 Division of Rheumatology, Immunology, and Allergy, Brigham and Women’s Hospital, Harvard Medical School, Boston, MA, USA; 5 Capio Artro Clinic, Stockholm, Sweden

**Keywords:** Gender differences, Femoral, Trauma, Sweden

## Abstract

**Background:**

A child who suffers a fracture or a soft-tissue injury at a young age faces an increased risk of subsequent injuries during childhood. This risk could be related to personal and family characteristics or to lower-than-average bone-mineral density. The purpose of this nationwide cohort study was to estimate the association between a femur shaft fracture at a young age and the subsequent risk of hospitalization for injuries during childhood.

**Methods:**

We compared the subsequent risk of hospitalization for injuries during childhood among 1,404 children (exposed) who were one to three years of age when they suffered a femur shaft fracture with the risk among 13,814 randomly selected, gender- and age-matched femur fracture–free children (unexposed). Hazard ratios (HRs) and 95% confidence intervals (CIs) for severe injuries defined as fractures or soft-tissue injuries requiring hospital admission were estimated in a Cox proportional hazards model.

**Results:**

Exposed children exhibited no significantly increased risk of upper-extremity fractures or soft-tissue injuries during childhood, regardless of sex and follow-up time. Boys exhibited a 162% increased risk of suffering a lower leg fracture requiring hospital admission (HR?=?2.62, 95% CI: 1.45–4.71), but the refracture risk was not significant for girls 2.02 (0.58–6.97).

**Conclusions:**

We found an increased risk for subsequent fractures in the lower leg that requires inpatient care during childhood for boys, but not for girls, who were one to three years of age when they first suffered a femur shaft fracture. This increased fracture risk is probably not simply the result of greater risk-taking among boys. The explanation might relate to factors affecting the bone quality of the lower leg.

## Background

The incidence of femur shaft fractures for boys and girls peaks among children aged one to three years, and the incidence is three times higher among boys than among girls [1,2]. Although all fracture types are more frequent in boys, such a great gender difference in this age group does not occur for other types of fractures [3,4]. The reasons for the difference are unknown, but studies examining both the behavior of children in this age group and parent-child interactions describe greater risk taking among boys and higher parental protectiveness toward girls [5].

Studies have also shown that a child with one hospital admission for an accident in the first five years of life runs an increased risk of experiencing another accident-related admission, compared with children of the same age and sex with no previous admissions for accidents [6-8]. An increased risk of repeated fractures could relate to the personal and family characteristics of these accident-prone children [9-11].

Data indicate that children without obvious metabolic bone diseases who experience their first fractures early in life are especially vulnerable to further fractures [3,12], perhaps owing to lower-than-average bone-mineral density [13-17]. Children aged one to three years who suffer a femur shaft fracture are usually treated with spica casting or traction for four weeks. This period of immobilization and no weight bearing, along with the associated inactivity, results in a loss of bone-mineral tissue and in muscle atrophy, which may influence the risk of further fractures [18-20].

The risk of further injuries demanding inpatient care among children who experience a severe fracture at young age has not, to our knowledge, previously been analyzed. The pediatric femur shaft fracture is a significant injury with a unique distribution of incidence regarding age and gender in one- to three-year-olds. In this study using Swedish databases, we hypothesized that a femur shaft fracture at this age is a predictor for further severe injuries during childhood and that fracture risk would differ between boys and girls.

## Methods

For the purpose of this population-based cohort study, we used data from the following three Swedish national registers: the Swedish Inpatient Register, the Swedish Medical Birth Registry, and the Cause of Death Registry. Record linkage of these registers was possible because a personal identification number is issued to every resident of Sweden [21].

We created the database, including a study cohort and a comparison cohort, using four steps. First, we identified children who suffered a femur shaft fracture between January 1, 1990, and December 31, 2005, in the Swedish Inpatient Register. The Swedish Inpatient Register contains information on all hospitalizations in Sweden, including each patient’s individual personal identification number, dates of hospitalizations, discharge code, and E-codes (external causes) according to the International Classification of Diseases (ICD-9: 1990 to 1996, ICD-10: 1997 to 2005) [22]. This study cohort examined the 5,124 children with femur shaft fractures considered in our recent paper on incidence and trends in femur shaft fractures [2].

Exposed individuals were defined as children who were one to three years of age when they suffered a *fracture of shaft of femur*, primary or secondary diagnostic code ICD: ICD-9/ICD-10: 821*/S723*. In ICD-9, fracture of femur, part unspecified, is included under diagnostic code 821*, and we chose therefore to include 15 children with ICD-10, diagnostic code *fracture of femur, part unspecified* (S72.9).

We excluded 56 children from the analysis with congenital medical conditions affecting the bone quality or the risk of trauma, regardless time of diagnosis. Exposed children (n?=?12) were censored if they had received a diagnosis that might affect their bone quality or risk of trauma—namely, bone tumor/cyst (malign or benign), epilepsy, and attention deficit/hyperactivity disorder (ADHD) (Table 1). If a child received a censoring diagnosis before the femur shaft fracture, the child was excluded from the analysis (n?=?6).

**Table 1:** Congenital medical conditions affecting the bone quality or the risk for injuries, diagnostic codes ICD-9/ICD-10

**Serious congenital medical conditions affecting bone quality**	**ICD-9**	**ICD-10**	**Number of exposed children**	**Number of unexposed children**
Osteogenesis imperfecta and Osteopetrosis	756.51, 756.52	Q78.0, Q78.2	23	0
Congenital malformations of the nervous system	740*–742*	Q00*–Q07*	17	24
Cerebral palsy and other paralytic syndromes	342*–344*	G80*–G83*	5	30
Pervasive developmental disorders	299*	F84*	3	16
Osteoporosis	733.0	M80*–M81*	3	0
Cystic fibrosis	277.0	E84*	2	2
Spinal muscular atrophy and related syndromes	335*	G12*	2	1
Hypopituitarism	253.3	E23.0	1	9
Down syndrome	758.0	Q90*	0	16
Marfan syndrome or Congenital malformation syndromes predominantly associated with short stature	759.81, 759.82	Q87.4*, Q87.1	0	5
Reduction defects of lower limb	755.3	Q72*	0	3
Muscular dystrophy	359.1	G71.0	0	3
Arthrogryposis multiplex congenita	754.89	Q74.3	0	1
Ehlers-Danlos syndrome	756.83	Q79.6	0	1
Total number of children with congenital medical conditions affecting bone quality			**56**	**111**
**Non-congenital medical conditions affecting bone quality or risk of trauma**				
Epilepsy	345*	G40*–G41*	4	23
Attention deficit/hyperactivity disorder	314*	F90*	2	8
Solitary bone cyst or Aneurysmal bone cyst	733.21, 733.22	M85.4, M85.5	2	1
Malignant neoplasm of lymphatic and hematopoietic tissue	200*–208*	C81*–C96*	1	13
Visual disturbances and blindness	368*-369*	H53*–H54*	1	9
Juvenile idiopathic arthritis	714.3*	M08*	1	3
Organ or tissue replaced by transplant	V42*	Z94*	1	0
Ulcerative colitis	556*	K51*	0	9
Benign neoplasm of long bones of lower limb	213.7	D16.2–D16.3	0	3
Malignant neoplasm of long bones of lower limb	170.7	C40.2–C40.9	0	0
Vitamin D deficiency	268*	E55.0, E55.9	0	0
Total number of children with non-congenital medical conditions affecting bone quality or risk of trauma			**12**	**69**

*Includes all forth and fifth positions.

**Figure 1:**
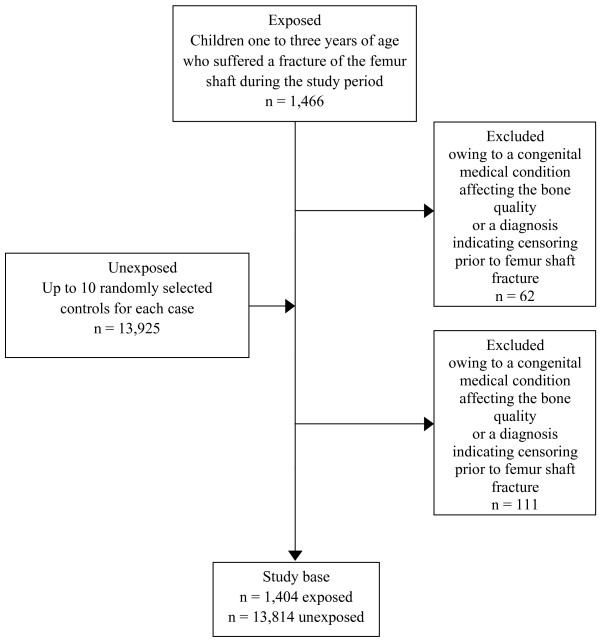
Flow chart of cases and matched controls.

Second, up to 10 unexposed children (median 10; range of unexposed per case 7–10) were randomly selected for each exposed child from the Swedish Medical Birth Registry, which includes data on practically all deliveries in Sweden [23]. Unexposed children were individually matched by sex, year of birth, and county of residence. The unexposed had no diagnosis of *fracture of femur* in the Swedish Inpatient Register, nor were they siblings of a child with a femur shaft fracture. Using the same definition as for the exposed group, we excluded from the unexposed group 111 children with histories of medical conditions that affect bone quality or risk of trauma. Unexposed children (n?=?69) were censored according to the same criteria applied to the exposed children. A total of 1,404 children and their 13,814 unexposed controls met the inclusion criteria (Figure 1).

Third, additional information regarding all hospital admissions since birth, until the age of 14 years or until December 31, 2005, whichever came first, was identified for exposed and unexposed children from the Swedish Inpatient Register. The rationale for the choice of age 14 is that patients in Sweden under the age of 15 are considered children regarding diagnoses of conditions caused by trauma.

Fourth, data were linked with the Cause of Death Registry in order to retrieve dates of death [24].

These two groups were compared to assess the risk of injury ICD-9/ICD-10: 800–848/S00–S99, T00–T14 (except for femur fractures, regardless of localization, ICD-9/ICD-10: 820–821/S72*). Diagnostic codes and E-codes (external causes) according to ICD-9 (E967, 995F, V61C) and ICD-10 (T74*, Y07*) were used to identify injuries caused by non- accidental trauma. To identify injuries classified as undetermined whether accidentally or purposely inflicted, diagnostic codes and E-codes according to ICD-9 (E988) and ICD-10 (Y33* and Y34*) were used. Only severe injuries defined as fractures or soft-tissue injuries (defined as all injury types except fractures) requiring hospital admission were identified because minor injuries are treated in outpatient settings. For the exposed, all injuries that occurred at the index date were not considered within the first 18 months of follow-up; these admissions were likely related to the injuries at index date and not to new injuries. We used the same washout period for the same injuries regarding the matched unexposed children.

The Stockholm Regional Ethical Review Board approved the study (Dnr2006/399–31).

### Statistical analysis

Descriptive statistics employed frequency and percentages. The two primary outcomes for this study were fracture and soft-tissue injury. The risk of having an outcome was calculated using Cox proportional hazards models and expressed as hazard ratios (HRs) with 95% confidence intervals (CIs). The hazard ratio was adjusted for year of fracture and the corresponding date for the unexposed, year of birth, and sex. The start of follow-up was defined as the date of the femur shaft fracture for exposed cases and as the corresponding date for the matched controls. Follow-up continued until the patient received an injury diagnosis or a diagnosis that excluded him or her from study, died, or reached age 15, or until December 31, 2005, whichever came first. A person could have more than one study endpoint (different injuries). Data were stratified by gender, and separate analyses examined fractures and soft-tissue injuries. Furthermore, fractures in upper extremities and in lower extremities (except for femur fractures, regardless of localization), were analyzed. The rationale for excluding femur fractures as an endpoint for the exposed children is that the unexposed had no diagnosis of *fracture of femur* in the Swedish Inpatient Register. In addition, the number of children with the most common type of upper limb fracture, lower limb fracture and soft tissue injury were reported. Multiple fractures and multiple soft-tissue injuries as well as injuries related to determined and undetermined non- accidental trauma were also stated. The assumption of proportional hazard was verified by comparing the difference in HR for follow-up time to injury between children with a follow-up period shorter than three years and children with a follow-up period of more than three years. No signs of insufficient proportionality were detected. An HR was considered significant if the 95% CI did not include 1.00. All statistical analyses were performed using SAS 9.3 for Windows (SAS Institute Inc., Cary, NC, USA) and IBM SPSS Statistics software, version 20 for Windows (SPSS Inc., Chicago, IL, USA).

## Results

Our cohort comprised 1,404 children, each exposed to a femur shaft fracture between the ages of one and three years (hereafter exposed children) and 13,814 matched controls (hereafter unexposed children). Five (three within 30 days after the femur shaft fracture) of the exposed children and 16 of the unexposed children died before the age of 15 years. We observed 97 children with injuries that required hospital admission among the exposed children during 12,234 person-years of follow-up (mean per child 8,7 years), compared to 885 injuries that required hospital admission among the unexposed children during 120,849 person-years of follow-up (mean per child 8,7 years).

The cohort characteristics, as well as rates of the different types of injuries, stratified by gender, are summarized in Table 2. The risk of injury for an exposed child was not higher than that among matched children with no history of femur shaft fracture (HR?=?1.08, 95% CI: 0.88–1.33) (n?=?97). When the type of injury was assessed in separate analyses, the risk of an injury resulting in a fracture was 38% higher among the exposed children (HR?=?1.38, 95% CI: 1.04–1.84) (n?=?54). However, the increased risk was seen only among boys, and it rose to 50% (HR?=?1.50, 95% CI: 1.10–2.03) (n?=?47). The association between a femur shaft fracture and future fractures was seen only in lower leg fractures (HR?=?2.49, 95% CI: 1.46–4.23) (n?=?17). This risk could only be linked to boys, who demonstrated a 162% increased risk (HR?=?2.62, 95% CI: 1.45–4.71) (n?=?14) of suffering a fracture in a lower limb that required hospital admission. The increased risk for boys was significant regardless of whether the lower leg fracture occurred within three years or more than three years after the exposure to a femur shaft fracture. The association between a femur shaft fracture and soft-tissue injuries was not significant regardless of gender and follow-up time (Table 3).

**Table 2:** Characteristics of the study subjects

**Variable**	**Exposed (N?=?1,404)**		**Unexposed (N?=?13,814)**	
	**Boys**	**Girls**	**Boys**	**Girls**
Total, N (%)	1,070 (76.2)	334 (23.8)	10,516 (76.1)	3,298 (23.9)
Number of children with injuries^a^	83 (7.8)	14 (4.2)	717 (6.8)	168 (5.1)
Number of children with fractures^b^	47 (4.4)	7 (2.1)	312 (3.0)	76 (2.3)
Number of children with fractures, upper limb^c^	29 (2.7)	4 (1.2)	239 (2.3)	58 (1.8)
Number of children with fracture of the lower end of radius or ulna^d^	13 (1.2)	0 (0.0)	90 (0.9)	21 (0.6)
Number of children with fractures, lower limb^b, c^	14 (1.3)	3 (0.9)	53 (0.5)	15 (0.5)
Number of children with fracture of shaft of tibia and fibula^e^	10 (0.9)	2 (0.6)	16 (0.2)	4 (0.1)
Number of children with soft-tissue injury	38 (3.6)	8 (2.4)	457 (4.3)	105 (3.2)
Number of children with intracranial injuries, the most common type of soft-tissue injury^f^	23 (2.1)	5 (1.5)	278 (2.6)	62 (1.9)
Number of children with multiple fractures^g^	9 (0.8)	1 (0.3)	42 (0.4)	14 (0.4)
Number of children with multiple soft-tissue injuries^g^	4 (0.4)	1 (0.3)	47 (0.4)	12 (0.4)
Number of children with injuries caused by non-accidental trauma	0 (0.0)	1 (0.3)	5 (0.0)	0 (0.0)
Number of children with injuries undetermined whether accidentally or purposely inflicted	0 (0.0)	0 (0.0)	2 (0.0)	0 (0.0)

^a^Injuries resulting in a fracture or soft-tissue injury requiring hospital admission. ^b^Except for femur fractures (regardless of localization). ^c^Some children had experienced both upper- and lower-limb fractures. ^d^The most common type of upper limb fracture. Diagnostic codes according to ICD-9 (813.4 – 813.5) and ICD-10 (S525*-526*). ^e^The most common type of lower limb fracture. Diagnostic codes according to ICD-9 (823.2 – 823.3) and ICD-10 (S822*). ^f^Diagnostic codes according to ICD-9 (850 - 854) and ICD-10 (S06*). ^g^*Multiple* is defined as involving more than one fracture or soft-tissue injury requiring hospital admission during the study period.

**Table 3:** Association between femur shaft fractures and injury requiring hospital admission grouped according to follow-up time after injury among 1,404 children (exposed) who were one to three years of age when they suffered a femur shaft fracture with the risk among 13,814 (unexposed) randomly selected, gender- and age-matched femur fracture–free children

	**Regardless of follow-up time**			**Follow-up, up to three years**			**Follow-up, more than three years**			**P-value**^**a**^
	**Number of events exposed**	**Number of events unexposed**	**HR**^**b **^**(CI)**	**Number of events exposed**	**Number of events unexposed**	**HR**^**b **^**(CI)**	**Number of events exposed**	**Number of events unexposed**	**HR**^**b **^**(CI)**	
All injuries^c^	97	885	1.08 (0.88–1.33)	23	241	0.94 (0.61–1.44)	74	644	1.13 (0.89–1.44)	0.45
Boys	83	717	1.14 (0.91–1.43)	22	199	1.09 (0.70–1.69)	61	518	1.16 (0.89–1.51)	0.80
Girls	14	168	0.91 (0.42–1.98)	1	42	0.23 (0.03-1.69)	13	126	1.02 (0.57–1.80)	0.16
All fractures	54	388	**1.38 (1.04–1.84)**	10	73	1.35 (0.70–2.62)	44	315	**1.39 (1.01–1.90)**	0.94
Boys	47	312	**1.50 (1.10–2.03)**	10	57	1.73 (0.88–3.38)	37	255	**1.44 (1.02–2.04)**	0.64
Girls	7	76	0.91 (0.42–1.98)	0	16	N.A.	7	60	1.15 (0.53–2.52)	0.98
Upper-limb fractures^d^	33	297	1.09 (0.76–1.57)	5	53	0.93 (0.37–2.32)	28	244	1.13 (0.76–1.67)	0.70
Boys	29	239	1.19 (0.81–1.75)	5	42	1.17 (0.46–2.95)	24	197	1.20 (0.79–1.83)	0.96
Girls	4	58	0.68 (0.25–1.86)	0	11	N.A.	4	47	0.83 (0.30–2.31)	0.98
Lower-limb fractures^d, e^	17	68	**2.49 (1.46–4.23)**	5	15	**3.27 (1.19–9.00)**	12	53	**2.26 (1.21–4.23)**	0.54
Boys	14	53	**2.62 (1.45–4.71)**	5	10	**4.90 (1.67–14.33)**	9	43	**2.08 (1.01–4.26)**	0.19
Girls	3	15	2.02 (0.58–6.97)	0	5	N.A.	3	10	3.05 (0.84–11.10)	0.99
Soft-tissue injury	46	562	0.80 (0.59–1.08)	13	171	0.74 (0.43–1.31)	33	391	0.83 (0.58–1.18)	0.77
Boys	38	457	0.81 (0.58–1.13)	12	145	0.81 (0.45–1.47)	26	312	0.81 (0.54–1.21)	0.99
Girls	8	105	0.76 (0.37–1.55)	1	26	0.38 (0.05–2.77)	7	79	0.89 (0.41–1.92)	0.43

^a^P-value for difference between HR up to three years and HR more than three years. ^b^Adjusted by matching for year of fracture and corresponding date for unexposed, age, and sex. ^c^Injuries resulting in a fracture or soft-tissue injury requiring hospital admission. ^d^Some children had experienced both upper- and lower-limb fractures. ^e^Except for femur fractures (regardless of localization). Significant values are written in bold type.

## Discussion

In this nationwide registry-based cohort study, we confirmed the previously known fact that there is an increased risk of a repeated fracture during childhood for a child who has been admitted to hospital for a fracture at a young age [3,12]. The novel aspect of this study is the retrospective analysis that allows us to test the hypothesis that the risk of hospitalization for injuries, both fractures and soft-tissue injuries during childhood, is influenced by a femur shaft fracture at young age and to determine whether fracture risk differs between boys and girls. Previous studies on repeated injuries have not evaluated soft-tissue injuries and fractures separately [6-8], and studies showing that an earlier fracture is associated with increased risk of new fractures during childhood have not included the risk of soft-tissue injuries in their analyses [3,12].

The incidence of femur shaft fractures among boys and girls peaks in the age group of one to three years, and the incidence rate ratio of boys to girls is 3:1 [2]. The reasons for the difference are unknown, but they probably relate to higher levels of risk taking among boys, which may correlate to a possible imbalance between demands placed on the femur and bone-mineral density [5,25].

Fracture risk, regardless of location and the child’s age, is higher among boys than among girls, and it has been suggested that a greater skeletal fragility relative to body size contributes to this gender difference [26]. A recent meta-analysis by Clark et al., concluded that children who experience fractures have lower bone-mineral density than children who do not experience fractures [27]. This concurs with the first study on the subject by Landin and Nilsson [14], who analyzed bone-mineral content in children with fractures. The patients were re-examined almost 30 years later, and the results showed that males with a fracture in childhood had a lower bone mass and smaller bone size at follow-up [28].

The present study found that boys who suffered a femur shaft fracture between the ages of one and three had an increased risk of lower leg fracture during childhood. Interestingly, there were no significantly increased risks for upper-limb fractures or soft-tissue injures for boys or girls. We can only speculate on the underlying explanations for the study findings, but the increased risk cannot be explained by simply pointing to higher levels of risk taking among boys or by positing that children with fractures generally have lower bone mass or slenderer bones than children without fractures do.

The literature has identified several risk factors for injuries in children, including inherited factors and lifestyle factors (e.g., nutritional factors and vigorous physical activity), as well as behavioral characteristics of the child, the family, and the social and physical environment [10,29]. The results of this study may be associated with factors affecting the bone strength of the lower legs. Immobilization and associated periods of inactivity are known to induce bone-mineral loss and muscle atrophy, and they affect the lower limb distal to the fracture site, thus influencing the risk of further fractures [18,19]. On the other hand, in a prospective study by Ceroni et al., examining bone mass in adolescents after a lower-limb fracture, a full bone recovery was seen after 18 months [20]. This contradicts our finding of an increased risk for boys regardless of whether the lower leg fracture occurred within three years or more than three years after the femur shaft fracture. There is, of course, a possibility that factors act together. For example, impaired bone strength of the lower limb after a femur shaft fracture may affect both girls and boys but increase the risk for subsequent lower limb fractures only among boys because of their greater tendency, to engage in risk-taking behavior.

There are limitations to our study. Yeh et al., found that only 13.4% of children with confirmed fractures were admitted to hospital, whereas 86.6% received outpatient care [12]. This is consistent with the results from the Swedish study by Hedström et al., who found that the overall fracture incidence for children ages 0–16 years was 208 per 100,000, compared to an incidence of admittances owing to fractures of 40 per 100,000 children [30]. Most childhood fractures affect the upper limbs, but lower limb fractures are to a greater degree associated with severe trauma that requires hospital admission. Because we did not have information on fractures treated in outpatient settings, we cannot directly compare our results with those of previous studies that found increased risk of repeated fractures among children [3,12]. On the other hand, previous studies on repeated childhood trauma deal with injuries regardless of whether they were benign or severe. In contrast, we examined injuries that required hospital admission—by definition, significant injuries. This is a registry-based study, and we did not have access to the charts or radiographs to confirm the diagnosis or side of each extremity injury. Therefore, some selection bias may have occurred. However, the quality of the Swedish Inpatient Register data has been systematically reviewed, and the accuracy of the coding is reported to be high [22]. We therefore believe that the present study likely includes all patients ages one to three who were hospitalized with femur shaft fractures in Sweden during the observation period. Non-accidential trauma (NAT) may be a confounder to the risk of subsequent fracture. Though, our method of using ICD-codes to identify undetermined intent or NAT probably resulted in an underestimation of the rate of physical abuse [31]. Another limitation is that we did not have information regarding body mass index. Obesity in children have been reported to be associated to an increased risk of lower leg fractures [32]. Although our group of unexposed children did not differ from the exposed children in age, gender, or county of residence, the unexposed children had no diagnosis of *fracture of femur* in the Swedish Inpatient Register. However, the intent of this analysis was to focus on new traumas and not on fractures at the same site (femur). Children were excluded from our study if they were diagnosed with ADHD since such children are more likely to suffer injury [33]. The rationale for not excluding them from the start of the study period is that the onset for ADHD varies; however, since we did not use information from prescriptions, there may be children in our study (in both the exposed and the unexposed group) with diagnosed or undiagnosed ADHD. It is possible that the experience of a femur shaft fracture at a young age changes children’s behavioral habits and potentially their parents’ attitudes toward childhood injury risk. This change in attitude could affect the likelihood that they would seek medical care for their child. However, the decision to admit a patient overnight is made by the treating doctor and not by parents, and this should minimize the risk of ascertainment bias.

This study includes hospital admissions for trauma over two decades. A fracture is a precise injury, and even if the treatment options for some fractures have changed during the study period, based on clinical experience we believe that the indication for fractures treated in inpatient care at the beginning and at the end of the study period are similar. In the previously mentioned study by Hedstrom et al., the incidence of fractures requiring admission increased by 38% between 1997 and 2007 in the northern part of Sweden [30]. Moreover, the threshold for admission for soft-tissue injuries has changed over time owing to new injury algorithms (e.g., computed tomography and head trauma) [34]. Hence, these factors will affect injury reporting in both the exposed and the unexposed children. In a previous study, we reported that sociodemographic variables influence the rate of femur shaft fractures, but we have not adjusted for this potential confounder [35].

This study provides valuable information regarding the risk of subsequent severe injuries during childhood. However, even though repeat accidents contribute little to the overall accident burden, additional studies are needed to better understand the bone health of children, especially boys, who suffer a femur shaft fracture at a young age.

## Conclusions

We found an increased risk for subsequent fractures in the lower leg that requires inpatient care during childhood for boys, but not for girls, who were one to three years of age when they first suffered a femur shaft fracture. This increased fracture risk is probably not simply the result of greater risk-taking among boys. The explanation might relate to factors affecting the bone quality of the lower leg.

## Abbreviations

HR: Hazard ratio; CI: Confidence intervals; ADHD: Attention deficit/hyperactivity disorder; ICD: International Classification of Diseases; NAT: Non-accidential trauma.

## Competing interests

The authors declare that they have no competing interests.

## Authors’ contributions

JvH had primary responsibility for study design, data analysis, statistics, writing, and manuscript editing. TS participated in study design, data analysis, statistics, writing, and manuscript editing. MI participated in study design, writing, and manuscript editing. AE participated in study design, writing, and manuscript editing. PMJ participated in study design, writing, and manuscript editing. All authors read and approved the final manuscript.

## Pre-publication history

The pre-publication history for this paper can be accessed here:

http://www.biomedcentral.com/1471-2431/14/62/prepub
